# Complete Genome Sequence of Escherichia coli ME8067, an Azide-Resistant Laboratory Strain Used for Conjugation Experiments

**DOI:** 10.1128/genomeA.00515-18

**Published:** 2018-06-21

**Authors:** Yasufumi Matsumura, Masaki Yamamoto, Satoshi Nakano, Miki Nagao

**Affiliations:** a Department of Clinical Laboratory Medicine, Kyoto University Graduate School of Medicine, Kyoto, Japan

## Abstract

We report here the complete genome sequence of Escherichia coli ME8067, an azide-resistant laboratory strain used for conjugation experiments. The ME8067 genome was closely related to E. coli strain K-12 substrain W3110. This genome sequence will support further genetic analysis of conjugative elements.

## GENOME ANNOUNCEMENT

Escherichia coli strain ME8067 (strain WD8014) is a laboratory E. coli strain that cannot ferment galactose due to its lack of UDP glucose pyrophosphorylase activity (1). This strain has been used for conjugation experiments as a recipient strain (2–4) because it is negative for fertility factors and is resistant to sodium azide. Recent advances in sequencing technology have enabled the sequencing of transconjugants, which contribute to a comprehensive understanding of transferable elements. Here, we present a complete genome sequence of E. coli ME8067, which is important for accurate nucleotide sequence analysis.

Whole-genome DNA was sequenced using both Illumina NextSeq 500 (150-bp paired-end) and Oxford Nanopore Technologies MinION (R9.4 flow cell) systems. A circular chromosome was obtained using a *de novo* hybrid assembly pipeline of Unicycler version 0.4.4 (5). The assembly resulted in average coverages of 186× with the NextSeq 500 system and 190× with the MinION system. The genome was annotated using the NCBI Prokaryotic Genome Annotation Pipeline (6).

The complete genome contains a 4,614,635-bp chromosome with a GC content of 50.8%, 4,413 coding sequences, 81 tRNA-coding genes, and 22 rRNA-coding operons. The Achtman multilocus sequence typing scheme (http://mlst.warwick.ac.uk/mlst/dbs/Ecoli) classified ME8067 into sequence type 10, which is a lineage of E. coli K-12 laboratory strains. Using the 41 E. coli K-12 genomes available on the NCBI assembly resource (https://www.ncbi.nlm.nih.gov/assembly) as of April 28, 2018, we constructed a phylogenetic tree based on core single-nucleotide polymorphisms (SNPs) with kSNP3 (7). ME8067 belonged to a cluster made up of the W3110 substrain and its derivatives (HMS174 and ZK126). This cluster was closely related to a cluster that included 15 MG1655 substrains.

Genomic comparison with the W3110 genome (GenBank accession no. AP009048) (8) using progressiveMauve (9) revealed that ME8067 had deletions totaling 53,580 bp, including *lacZYA*, IS*5* (6 copies), IS*2* (3 copies), and tRNAs. Deficiency of the *lac* operon can explain the lactose-nonfermenting characteristic of ME8067. Insertions totaling 34,648 bp included IS*5* (3 copies), tRNAs, and RtT small RNAs (sRNAs). Among the 10 prophages present in MG1655 (GenBank accession no. U00096), CPZ-55 was absent in W3110 but present in ME8067. All of the other 9 prophages were present in W3110, though ME8067 had a partial deletion of CP4-6, an insertion of IS*3* to DLP12, and an inversion of an internal segment within e14. Single-nucleotide polymorphisms (SNPs) and small nucleotide insertions/deletions (indels) were sought using SNIT (10) and annotated using snpEff (11). Between the W3110 and ME8067 genomes, 693 SNPs and 30 indels were present, resulting in 406 nonsynonymous mutations. An SNP in the *secA* gene resulting in the amino acid alteration A112T was found and may be associated with azide resistance (12). Another SNP in *galU* (amino acid alteration, P14S) may be associated with the lack of UDP glucose pyrophosphorylase activity.

The complete genome sequence of strain ME8067 revealed that it belongs to a lineage of E. coli strain K-12 substrain W3110 and will aid in comprehensive genetic analysis for conjugation experiments.

### Accession number(s).

The complete genome sequence of the chromosome of E. coli ME8067 has been deposited at GenBank under accession no. CP028703.
